# Supplementary material for: Self-activated superhydrophilic green ZnIn2S4 realizing solar-driven overall water splitting: close-to-unity stability for a full daytime
Source: Nat Commun. 2023 Nov 24;14:7676. doi: 10.1038/s41467-023-43331-x (PMC10667227; doi:10.1038/s41467-023-43331-x)
Supplement: Supplementary file 1 — Supplementary Information [file 41467_2023_43331_MOESM1_ESM.pdf]

Supplementary Information

Self-activated superhydrophilic green $ZnIn_2S_4$ realizing solar-driven overall water splitting: Close-to-unity stability for a full daytime

Wei-Kean Chong,[1] Boon-Junn Ng,[1] Yong Jieh Lee,[1] Lling-Lling Tan,[1] Lutfi Kurnianditia Putri,[1] Jingxiang Low,[1,2] Abdul Rahman Mohamed,[3] and Siang-Piao Chai[1,]*

[1] *Multidisciplinary Platform of Advanced Engineering, Department of Chemical Engineering, School of Engineering, Monash University Malaysia, Jalan Lagoon Selatan, 47500 Bandar Sunway, Selangor, Malaysia*

[2] *Department of Applied Chemistry, University of Science and Technology of China (USTC), 96 Jinzhai Road, Hefei, Anhui 230026, PR China*

[3] *School of Chemical Engineering, Universiti Sains Malaysia, 14300 Nibong Tebal, Pulau Pinang, Malaysia*

* Corresponding author. Email: chai.siang.piao@monash.edu

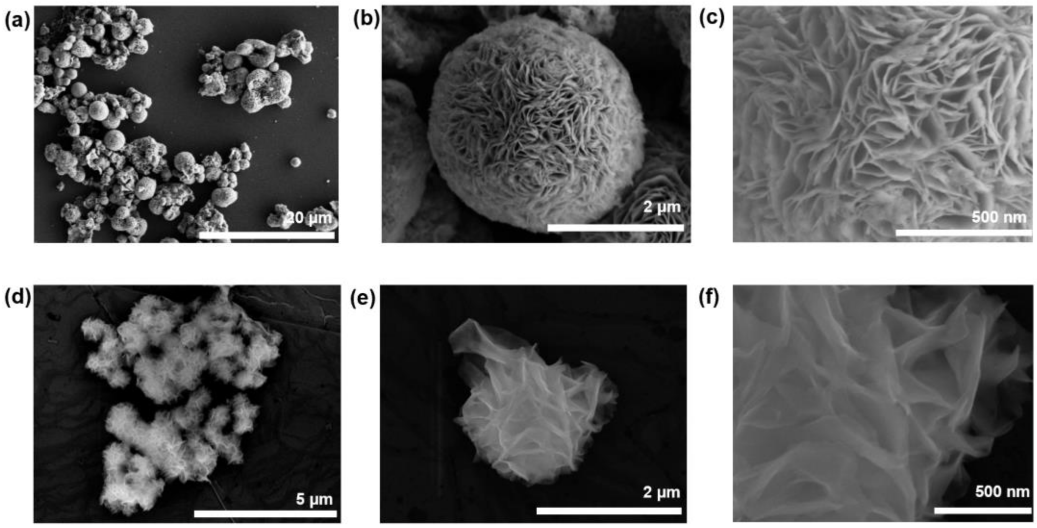

Supplementary Fig. 1. FESEM images with different magnification views of (a-c) ZIS and (d-f) gZIS.

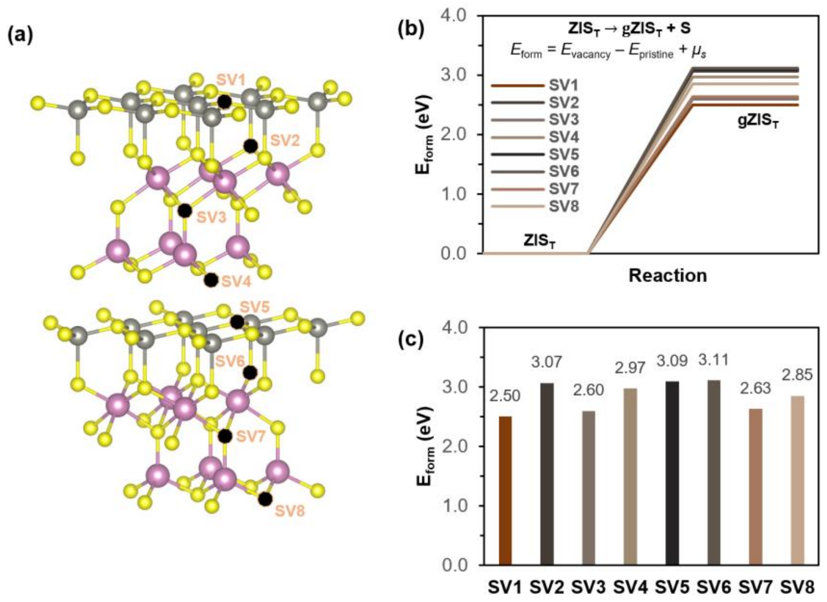

Supplementary Fig. 2. (a) Different potential sulfur vacancy positions on ZIS structure. Graphical representation of the computed E_{form} in (b) line graph along reaction pathway, and (c) bar chart comparing across different sulfur vacancy positions. ZIS_T and $gZIS_T$ denote the theoretical ZIS and gZIS structures, respectively.

The formation energy (E_{form}) was computed according to:

$$E_{form} = E_{vacancy} - E_{pristine} + \mu_s \quad \text{(S1)}$$

in which $E_{vacancy}$ is the free energy of the sulfur vacant ZIS and $E_{pristine}$ represents the free energy of the pristine ZIS. μ_s dictates the chemical potential of S atom removed from the bulk phase. The lowest E_{form} corresponds to the most energetically favorable structure.[1]

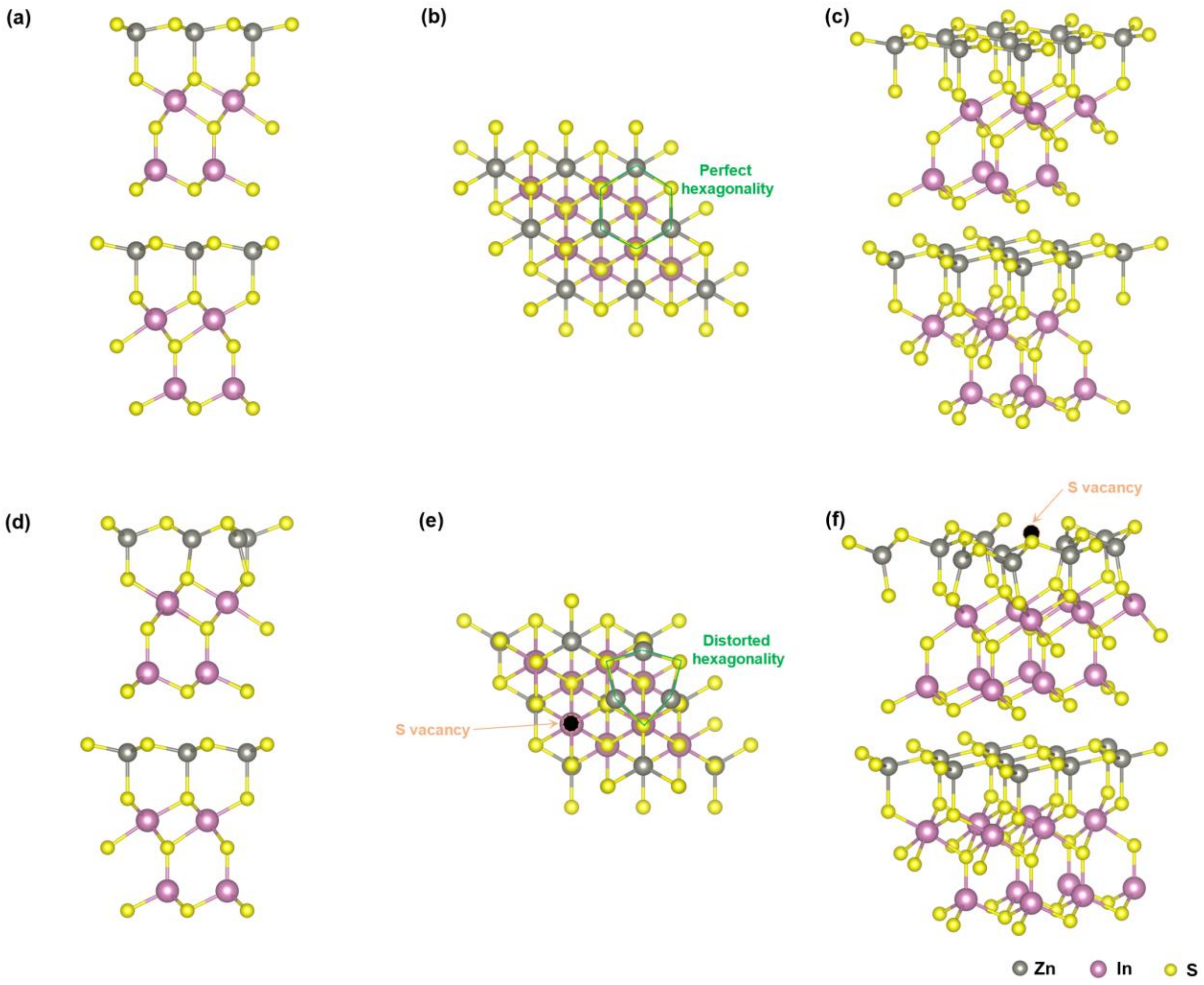

Supplementary Fig. 3. Theoretical structures of (a-c) pristine ZIS_T, and (d-f) sulfur-vacant $gZIS_T$ at different viewpoints.

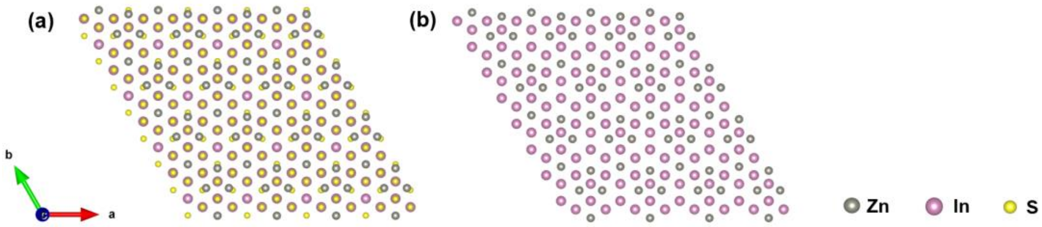

Supplementary Fig. 4. Simulated atomic arrangement in $gZIS_T$ along (001) facet with S atom (a) toggled-on, and (b) toggled-off.

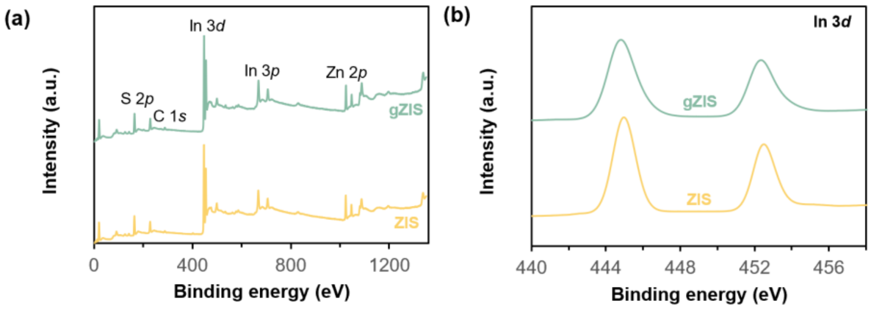

Supplementary Fig. 5. (a) XPS survey scan and (b) In 3*d* narrow scan for ZIS and gZIS.

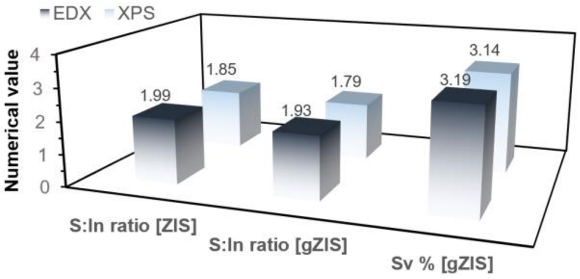

Supplementary Fig. 6. Comparison between EDX and XPS analyses on the S-to-In atomic ratio in ZIS and gZIS. The S_v % for both the analyses was also computed by taking In as the base of comparison following Supplementary Table 1.

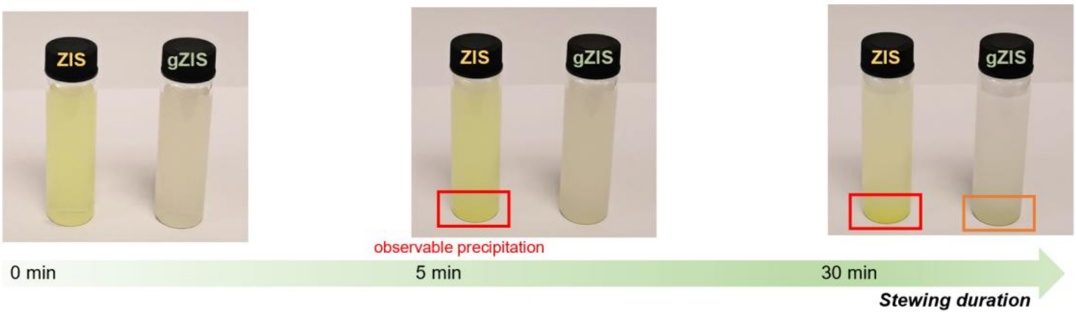

Supplementary Fig. 7. Water dispersion test for ZIS and gZIS in deionized water (concentration: 0.25 mg·mL^{-1})

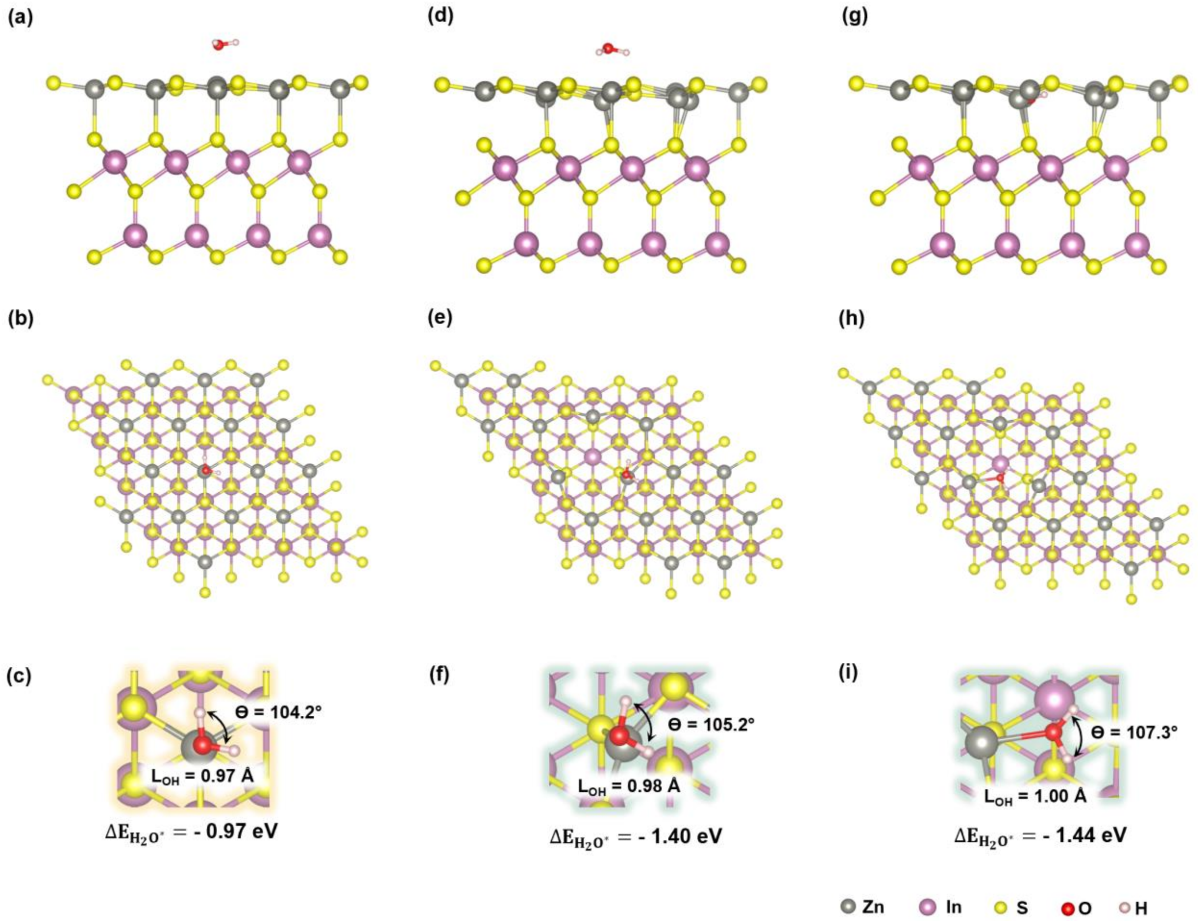

Supplementary Fig. 8. Different plane views of the water interaction along the basal plane at different locations: (a-c) on the Zn atom of ZIS_T, (d-f) on the Zn atom of $gZIS_T$, and (g-i) in the S_v position of $gZIS_T$.

The water adsorption energy is calculated by:[2]

$$E_{H_2O^*} = E_{PC+H_2O} - E_{PC} - E_{H_2O} \quad \text{(S2)}$$

whereby E_{PC+H_2O}, E_{PC} and E_{H_2O} dictate the total energy of a water molecule adsorbed onto the photocatalyst surface, surface energy of the photocatalysts and the energy of a free water molecule.

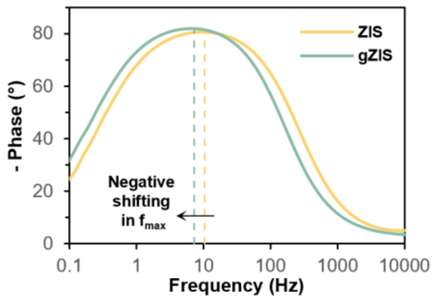

Supplementary Fig. 9. Bode-phase plot of ZIS and gZIS.

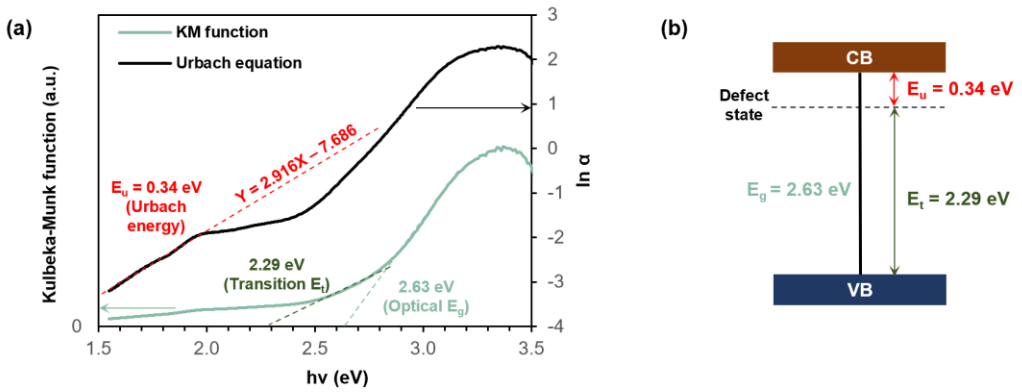

Supplementary Fig. 10. (a) Tauc plot and Urbach plot, as well as (b) schematic of defect state position within the band gap of gZIS.

The Urbach energy (E_u), corresponding to the impurity level below CB, is calculated following the Urbach equation:[3]

$$\alpha = \alpha_0 \exp\left(\frac{h\nu}{E_u}\right) \quad \text{(S3)}$$

which can be further linearized into:

$$\ln \alpha = \left(\frac{1}{E_u}\right) h\nu + \ln(\alpha_0) \quad \text{(S4)}$$

where α is the absorption coefficient, α_0 is the Urbach constant, hν is the incident photon energy and E_u is the Urbach energy (which can be calculated as the reciprocal of the gradient in the linearized Urbach equation). Consistent calculation is performed by extrapolating the transition energy from Tauc plot to determine the location of defect state as displayed in Supplementary Fig. 10a. It can be shown that both calculations, Urbach energy and transition energy, converge to the same position of the defect level, implying an accurate finding is achieved.

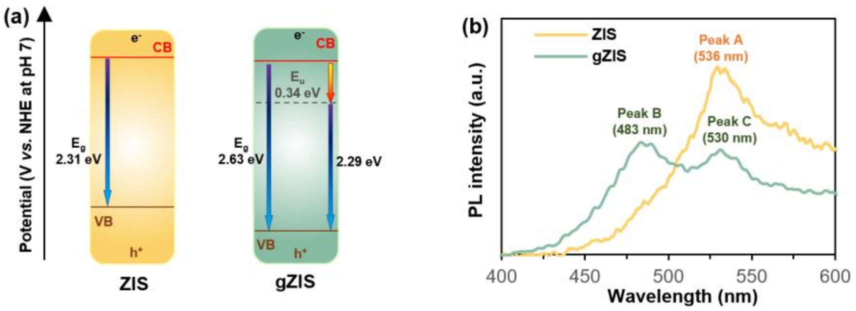

Supplementary Fig. 11. (a) Band structures and (b) PL spectra for ZIS and gZIS sample.

As presented in Supplementary Fig. 11, pristine ZIS exhibits a singular PL peak at 536 nm (approximately 2.31 eV), corresponding to its E_g determined through the KM relationship. Conversely, gZIS demonstrates two distinct PL peaks, in which the first peak at 483 nm (around 2.57 eV) arises from the intrinsic band-to-band radiative transition of photoexcited electrons from the CB to the VB, closely aligning with the E_g derived. The second peak at 530 nm (approximately 2.34 eV) originates from the extrinsic sub-band defect state introduced by S_v to the ground state. This value closely corresponds to the defect energy calculated using the Urbach's equation and transition energy.

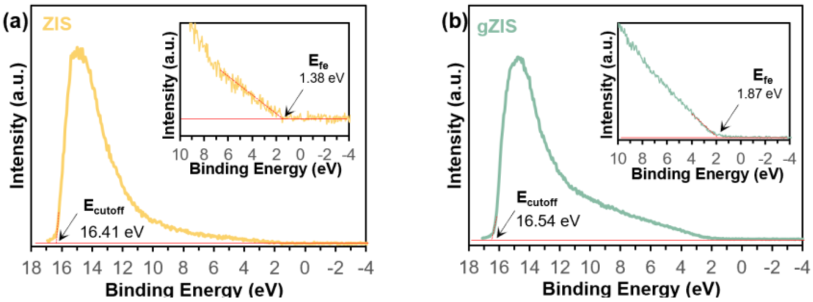

Supplementary Fig. 12. UPS spectra of (a) ZIS and (b) gZIS for determining the valence band energy.

The valence band energy (E_{VB}) with respect to vacuum was evaluated according to the formula:[4, 5, 6, 7]

$$E_{VB} = h\nu - (E_{cut} - E_{fe}) \quad \text{(S5)}$$

whereby hν represents the incident photon energy of He light source of 21.22 eV, E_{cut} denotes the electron cut-off edge, and E_{fe} is the Fermi edge of the samples. Following that, unit conversions were applied based on the relationship between vacuum energy (E_{vac}) and NHE potential (E_{NHE}) as in 0 V *vs.* NHE is equal to -4.44 eV in vacuum, as well as a pH correction factor of 0.059 pH to convert to NHE scale at pH 7. In short, ZIS possesses an E_{VB} of 6.19 eV below vacuum (1.34 V *vs.* NHE at pH 7) and gZIS exhibits an E_{VB} position of 6.55 eV below vacuum (1.70 V *vs.* NHE at pH 7).

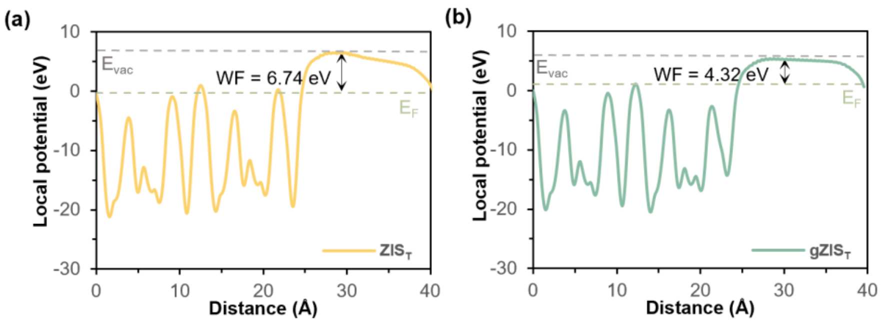

Supplementary Fig. 13. Theoretical work function (WF) of (a) ZIS_T and (b) $gZIS_T$.

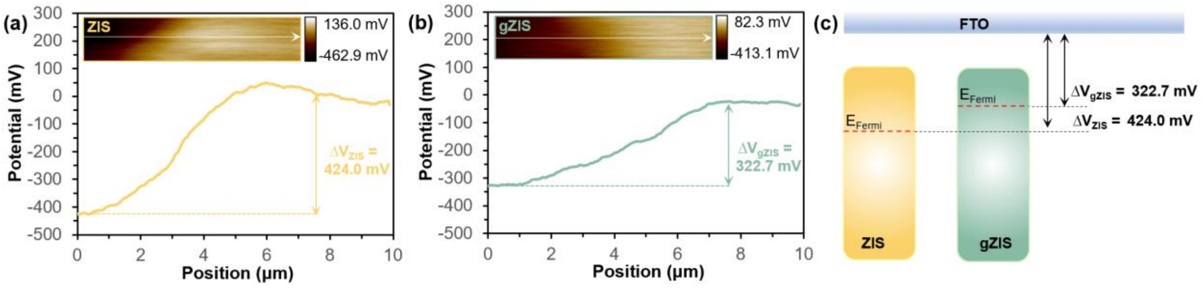

Supplementary Fig. 14. Potential line profiles of (a) ZIS and (b) gZIS, with insets showing the respective x-y scan area. The white line indicates the longitudinal scan direction. (c) Illustration of the estimated WF positions of ZIS and gZIS with respect to FTO.

The relative work function (WF) of the samples can be estimated by measuring the contact potential difference (CPD) between the sample and conductive reference (*i.e.*, FTO) across the interfacial boundary. As presented in Supplementary Fig. 14, the CPD values were measured by sweeping through the sample with a biased AFM probe, by which the counter bias voltage used in neutralizing the electric field was recorded. In this regard, gZIS possesses a comparatively lower CPD value (ΔV = 322.7 mV) than that of ZIS (ΔV = 424.0 mV). By taking FTO as a conductive reference, the local variation of WF in the samples could be attained and the changes in relative WF of the samples could be feasibly compared.[8] Thus, it could be observed that gZIS experiences reduction in WF, accompanied by the uplift of Fermi level to facilitate photogenerated electron transition.

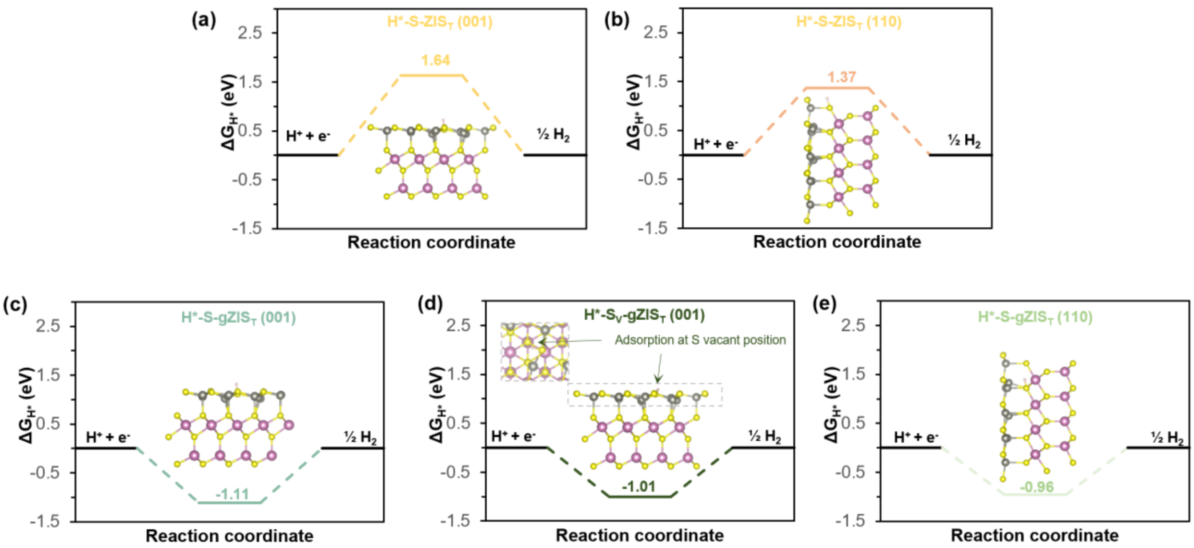

Supplementary Fig. 15. Free energy diagram for HER for (a) S atom at [001] facet of ZIS_T, (b) S atom at [110] facet of ZIS_T, (c) S atom at [001] facet of $gZIS_T$, (d) S_v position at [001] facet of $gZIS_T$, and (e) S atom at [110] facet of $gZIS_T$.

For single HER reaction, the corrected H^* adsorption Gibbs free energy (ΔG_{H*}) at U = 0 could be calculated via :[9]

$$\Delta G_{H*} = \Delta E_{H*} + (\Delta ZPE - T\Delta S)_{H^*} = \left(E_{H*} - E^* - \frac{1}{2}E_{H_2}\right) + 0.24 \quad (S6)$$

by which ΔE_{H*} is the differential adsorption energy of H^* on surface slab, E^* is the clean surface energy and E_{H_2} is the energy of free H_2.

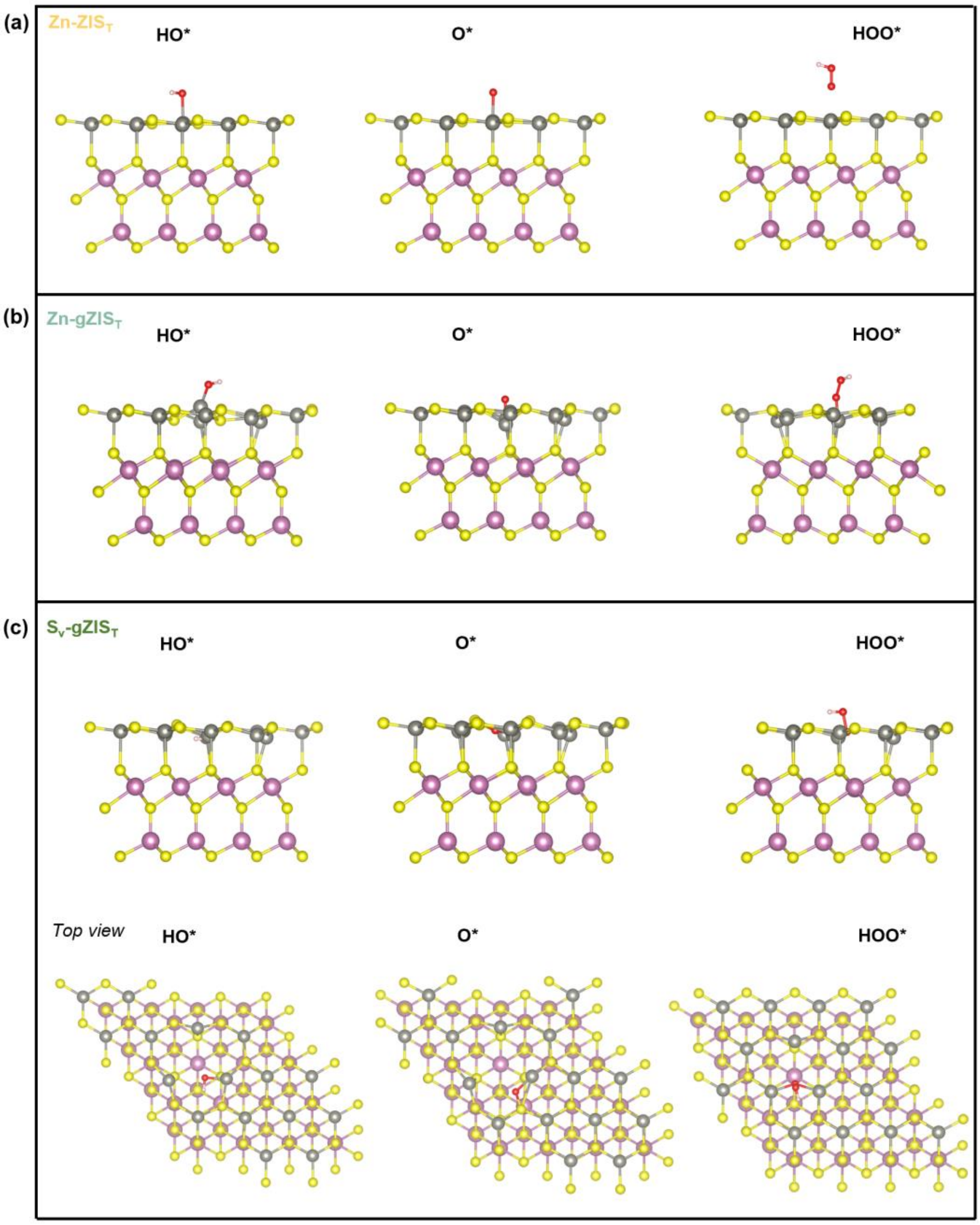

Supplementary Fig. 16. Optimized structural model of adsorbed HO*, O* and HOO* onto (a) Zn-ZIS$_T$, (b) Zn-gZIS$_T$ and (c) S$_V$-gZIS$_T$.

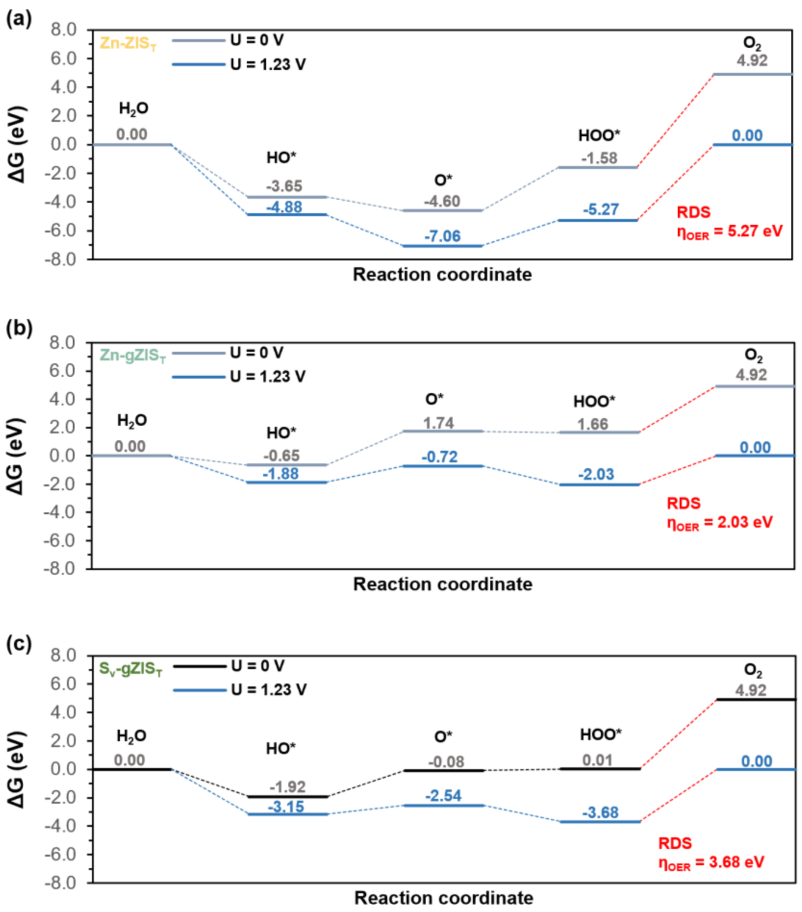

Supplementary Fig. 17. Free energies of (a) Zn-ZIS$_T$, (b) Zn-gZIS$_T$ and (c) S$_v$-gZIS$_T$ with U = 0 [no applied bias] and U = 1.23 V [standard equilibrium potential of OER]. Rate determining step (RDS) is marked as red in each of the sub-figure.

The OER process could be evaluated following the four elementary steps below which HO*, O* and HOO* adsorbed intermediate onto active surface slab (*).

$$* + H_2O \rightleftharpoons HO^* + H^+ + e^- \tag{S7}$$

$$HO^* \rightleftharpoons O^* + H^+ + e^- \tag{S8}$$

$$O^* + H_2O \rightleftharpoons HOO^* + H^+ + e^- \tag{S9}$$

$$HOO^* \rightleftharpoons * + O_2 + H^+ + e^- \tag{S10}$$

Thus, the respective Gibbs free energy of each step could be evaluated by referring to standard Gibbs free energy of water splitting ($\Delta G_{2H_2O \rightarrow O_2 + 2H_2}$) of 4.92 eV,[2] which is summarized as:

$$\Delta G_{OER1} = E_{HO^*} + \frac{1}{2}E_{H_2} - E_{H_2O} - E^* + (\Delta ZPE - T\Delta S)_{OER1} - eU \tag{S11}$$

$$\Delta G_{OER2} = E_{O^*} + \frac{1}{2}E_{H_2} - E_{HO^*} + (\Delta ZPE - T\Delta S)_{OER2} - eU \tag{S12}$$

$$\Delta G_{OER3} = E_{HOO^*} + \frac{1}{2}E_{H_2} - E_{O^*} - E_{H_2O} + (\Delta ZPE - T\Delta S)_{OER3} - eU \tag{S13}$$

$$\Delta G_{OER4} = \left(4.92 + 2E_{H_2O} - \frac{3}{2}E_{H_2}\right) + E^* - E_{HOO^*} + (\Delta ZPE - T\Delta S)_{OER4} - eU \tag{S14}$$

in which E_{HO^*}, E_{O^*} and E_{HOO^*} dictate the energy of surfaces with adsorbed HO*, O* and HOO*, respectively. The OER overpotential (η_{OER}) is defined as:

$$\eta_{OER} = \frac{\max(|\Delta G_{OER1}|,|\Delta G_{OER2}|,|\Delta G_{OER3}|,|\Delta G_{OER4}|)}{e}\Big|_{U=1.23\ V} \tag{S15}$$

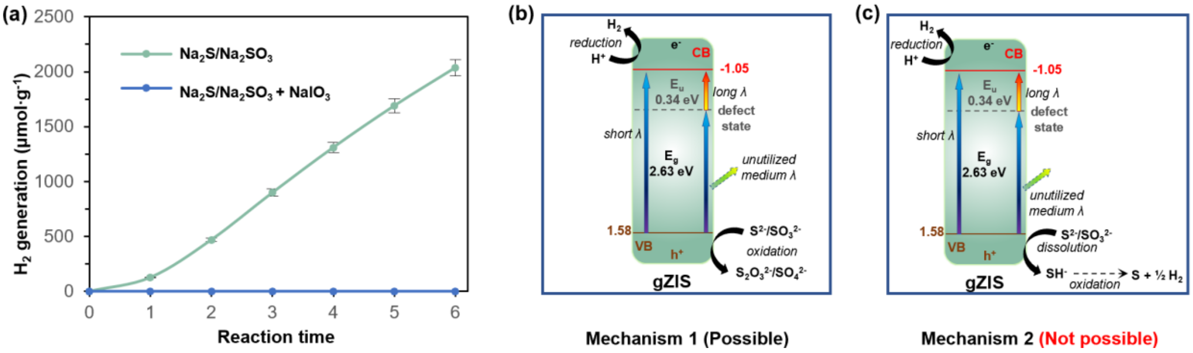

Supplementary Fig. 18. (a) Time-dependent solar-driven H_2 evolution rate of gZIS under two conditions: [1] Na_2S/Na_2SO_3, and [2] Na_2S/Na_2SO_3 + $NaIO_3$ sacrificial conditions. Error bars represent the standard deviation from two independent runs. Illustration of the different oxidations of S^{2-}/SO_3^{2-} where (b) irreversible oxidation to form $S_2O_3^{2-}/SO_4^{2-}$ – possible mechanism, and (c) oxidative production of H_2 – not possible mechanism.

It was previously shown that the potential oxidative production of H_2 from S^{2-} hole scavengers following the two-step mechanisms of dissolution and oxidation:[10]

$$S^{2-} + H_2O \rightleftharpoons SH^- + OH^- \quad (S16)$$

$$SH^- + h^+ \rightarrow S + \frac{1}{2}H_2 \quad (S17)$$

in which the oxidative production of H_2 involves dissolution of S^{2-} to form bisulfide ion (SH^-) and further oxidized by the holes in the VB of the semiconductor to produce H_2.

A control experiment was performed to validate any potential of the above-mentioned oxidative production of H_2, whereby additional 0.1 M of $NaIO_3$ was introduced into the system as electron scavenger to consume the photogenerated electrons. As evident in Supplementary Fig. 18 , there is no observable H_2 production in Na_2S/Na_2SO_3 + $NaIO_3$. This observation implies that the measured H_2 under Na_2S/Na_2SO_3 sacrificial conditions is in fact brought by the photoreduction of H^+ from the electron of the semiconductor (gZIS).

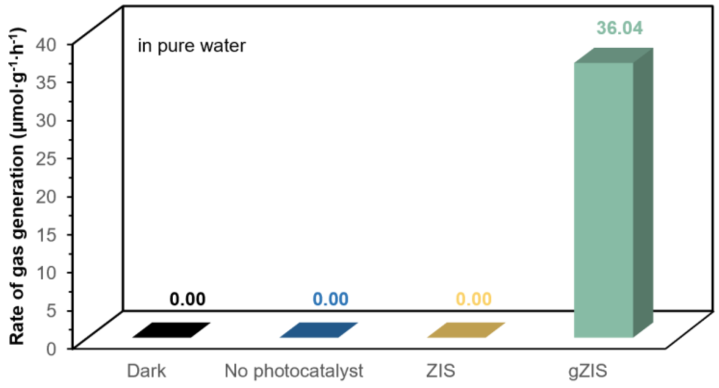

Supplementary Fig. 19. Control experiments performed for solar-driven pure water splitting.

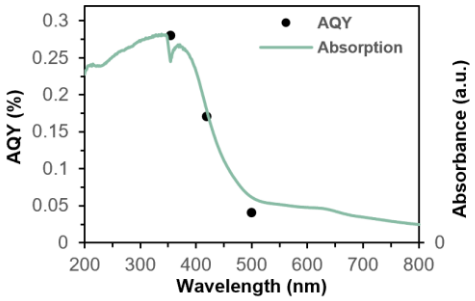

Supplementary Fig. 20. Wavelength-dependent AQY and UV-Vis DRS plot of gZIS in pure water.

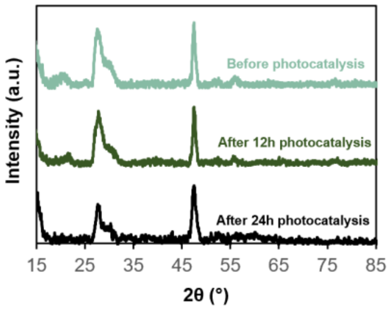

Supplementary Fig. 21. Comparison of XRD pattern of gZIS before and after overall solar-driven pure water splitting.

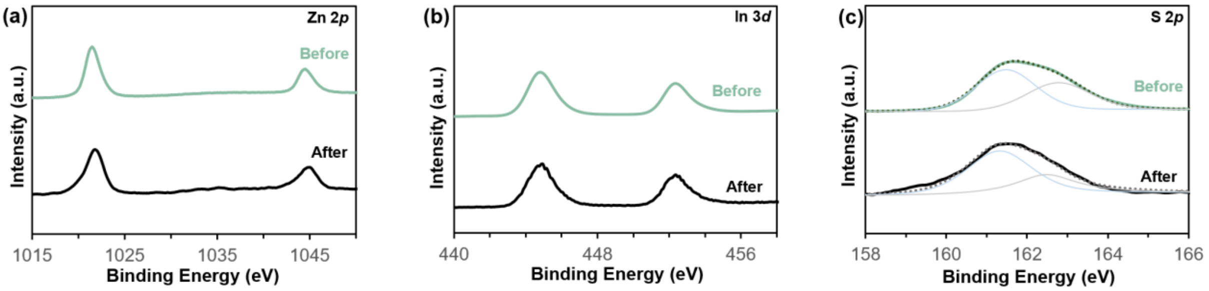

Supplementary Fig. 22. Comparison of XPS peak of gZIS before and after 24h overall solar-driven pure water splitting.

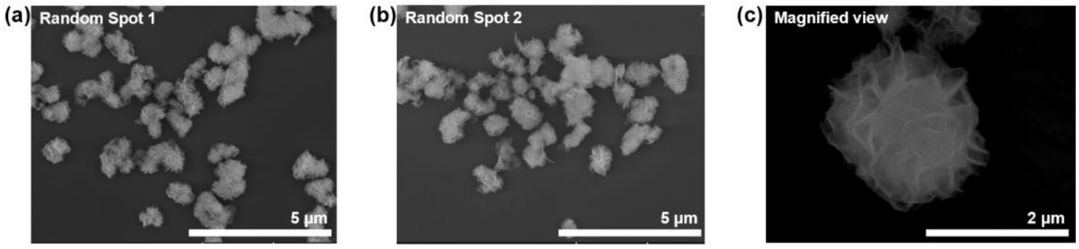

Supplementary Fig. 23. FESEM images of spent gZIS after 24h overall solar-driven pure water splitting, (a-b) random-spot overviews and (b) magnified image.

Supplementary Table 1. Atomic percentage of respective elements from EDX analysis, derived empirical formula and the percentage of sulfur vacancy concentration for ZIS and gZIS.

Structure	Zn %	In %	S %	Empirical Formula	S_v percent. (%)[a]
**ZIS**	15.10	28.35	56.55	$Zn_{1.07}In_{2.00}S_{3.99}$	-
**gZIS**	15.38	28.87	55.75	$Zn_{1.07}In_{2.00}S_{3.86}$	3.19

[a] The percentage of sulfur vacancy concentration (S_v percent.) is calculated by referring to the S-to-In atomic ratio in the structure as followed:[11]

$$S_v \text{ percent.} = \frac{\text{S:In}_{\text{ZIS}} - \text{S:In}_{\text{gZIS}}}{\text{S:In}_{\text{ZIS}}} \times 100\% \quad \text{(S18)}$$

where S:In_{ZIS} and $\text{S:In}_{\text{gZIS}}$ dictate the S-to-In atomic ratio in ZIS and gZIS, respectively.

Supplementary Table 2. Compilation of the EIS fitted parameters for samples under illumination.

Structure	R_{CT} (kΩ)	R_S (Ω)	CPE (µF)	Goodness of Fit
ZIS	31.36	9.9	23.34	0.998
gZIS	27.46	10.47	29.33	0.994

Supplementary Table 3. Biexponential fitting parameters for the samples.[a]

Structure	A_1 (%)	τ_1 (ns)	A_2 (%)	τ_2 (ns)	τ_{ave} (ns)	Goodness of Fit
ZIS	83.60	1.76	16.40	9.44	5.69	0.998
gZIS	87.89	1.50	12.11	17.41	11.28	0.993

[a] The TRPL decay curve is fitted following a biexponential kinetic model as follow:

$$I = A_1 e^{-\frac{t}{\tau_1}} + A_2 e^{-\frac{t}{\tau_1}} \quad \text{(S19)}$$

The average lifetime of the samples is computed by:

$$\tau_{ave} = \frac{A_1\tau_1^2 + A_2\tau_2^2}{A_1\tau_1 + A_2\tau_2} \quad \text{(S20)}$$

whereby τ_1 and A_1 represent the short fluorescent lifetime and its relative amplitude, whereas the τ_2 and A_2 dictate the long fluorescent lifetime and its relative amplitude.

Supplementary Table 4. Comparison of AQY performance (half-reaction) at 420 nm monochromatic light for gZIS with other recently reported conventional cocatalyst loaded or ZIS-based heterostructure.

Catalysts	Condition	AQY (%)	Ref.
**gZIS**	Na_2S/Na_2SO_3 (0.35M)	5.34	This work
Pt@CNT/ZIS	TEOA (10 vol%)	2.30	12
Pt@CQD/ZIS	TEOA (10 vol%)	2.30	12
Pt@ZIS	Na_2S/Na_2SO_3 (0.25/0.35M)	4.11	13
Pt@RGO/ZIS	TEOA (10 vol%)	4.40	12
Pt@$BiVO_4$/ZIS	TEOA (10 vol%)	4.23	14
Pt@Co-P/ZIS	Lactic acid (10 vol%)	4.30	15
Pt@Cu-ZIS	Ascorbic acid (0.1 M)	4.76	16
Pt@$CoFe_2O_4$/ZIS	TEOA (20 vol%)	5.00	17
Pt@Ti_3C_2TX/ZIS	TEOA (10 vol%)	11.14	18
gC_3N_4/ZIS	TEOA (10 vol%)	0.28	19
ZIS/In_2O_3	TEOA (15 vol%)	1.23	20
MoS_2/ZIS	Lactic acid (10 vol%)	3.08	21
WS_2/ZIS	Lactic acid (20 vol%)	3.20	22
NH_2-MIL-125(Ti)/ZIS	Na_2S/Na_2SO_3 (0.25/0.35M)	4.30	23
$Ni_{1-x}Co_xSe_2$/ZIS	TEOA (20 vol%)	4.35	24
S-NH_2-UiO-66/ZIS/MoS_2	TEOA (10 vol%)	4.95	25
Co-N-CN/ZIS	TEOA (15 vol%)	5.07	26
Ni_2P/ZIS	Lactic acid (10 vol%)	7.70	27
SnSe/ZIS	TEOA (10 vol%)	9.00	28
MIL-101/ZIS	Lactic acid (10 vol%)	10.93	29

Supplementary Table 5. Comparison of AQY at 420 nm monochromatic light and STH performance (pure water) for gZIS with other single-component sulfide-based photocatalysts.

Catalysts	AQY (%)	STH (%)	Ref.
**gZIS**	0.17	0.002	This work
Rhombohedral ZIS modified with Pt and Cr cocatalysts	0.041	0.021	30
P-$Zn_{0.5}Cd_{0.5}S_{1-x}$	0.15	-	3
P-ZIS	0.16	-	31
Ag-ZIS	0.57	0.003	32
$Y_2Ti_2O_5S_2$ modified with IrO_2 and Rh/Cr_2O_3 cocatalysts	0.36	0.007	33
CdS modified with Pt and Cr_2O_3 cocatalysts	0.22 (430 nm)	-	34

Supplementary References

1. Shi X, *et al.* Inert basal plane activation of two-dimensional $ZnIn_2S_4$ via Ni atom doping for enhanced co-catalyst free photocatalytic hydrogen evolution. *J. Mater. Chem. A* **8**, 13376-13384 (2020).

2. Zhou X, Dong H, Ren A-M. Exploring the mechanism of water-splitting reaction in NiO_x/β-Ga_2O_3 photocatalysts by first-principles calculations. *Phys. Chem. Chem. Phys.* **18**, 11111-11119 (2016).

3. Ng B-J, Putri LK, Kong XY, Pasbakhsh P, Chai S-P. Overall pure water splitting using one-dimensional P-doped twinned $Zn_{0.5}Cd_{0.5}S_{1-x}$ nanorods via synergetic combination of long-range ordered homojunctions and interstitial S vacancies with prolonged carrier lifetime. *Appl. Catal., B* **262**, 118309 (2020).

4. Wang H, *et al.* Highly active deficient ternary sulfide photoanode for photoelectrochemical water splitting. *Nat. Commun.* **11**, 3078 (2020).

5. Liu D, *et al.* In situ constructing atomic interface in ruthenium-based amorphous hybrid-structure towards solar hydrogen evolution. *Nat. Commun.* **14**, 1720 (2023).

6. Zhao D, *et al.* Boron-doped nitrogen-deficient carbon nitride-based Z-scheme heterostructures for photocatalytic overall water splitting. *Nature Energy* **6**, 388-397 (2021).

7. Fabian DM, Ardo S. Hybrid organic–inorganic solar cells based on bismuth iodide and 1,6-hexanediammonium dication. *J. Mater. Chem. A* **4**, 6837-6841 (2016).

8. Xu D, Zhang S-N, Chen J-S, Li X-H. Design of the synergistic rectifying interfaces in mott–schottky catalysts. *Chem. Rev.* **123**, 1-30 (2023).

9. Zhai P, *et al.* Engineering active sites on hierarchical transition bimetal oxides/sulfides heterostructure array enabling robust overall water splitting. *Nat. Commun.* **11**, 5462 (2020).

10. Kamat PV, Jin S. Semiconductor photocatalysis: "Tell Us the Complete Story!". *ACS Energy Lett.* **3**, 622-623 (2018).

11. Zhang S, *et al.* Gradient hydrogen migration modulated with self-adapting S vacancy in copper-doped $ZnIn_2S_4$ nanosheet for photocatalytic hydrogen evolution. *ACS Nano* **15**, 15238-15248 (2021).

12. Xia Y, Li Q, Lv K, Tang D, Li M. Superiority of graphene over carbon analogs for enhanced photocatalytic H_2-production activity of $ZnIn_2S_4$. *Appl. Catal., B* **206**, 344-352 (2017).

13. Tian F, Zhu R, Song K, Niu M, Ouyang F, Cao G. The effects of hydrothermal temperature on the photocatalytic performance of $ZnIn_2S_4$ for hydrogen generation under visible light irradiation. *Mater. Res. Bull.* **70**, 645-650 (2015).

14. Hu J, Chen C, Zheng Y, Zhang G, Guo C, Li CM. Spatially separating redox centers on Z-scheme $ZnIn_2S_4$/$BiVO_4$ hierarchical heterostructure for highly efficient photocatalytic hydrogen evolution. *Small* **16**, 2002988 (2020).

15. Liu Q, Wang M, He Y, Wang X, Su W. Photochemical route for synthesizing Co–P alloy decorated $ZnIn_2S_4$ with enhanced photocatalytic H_2 production activity under visible light irradiation. *Nanoscale* **10**, 19100-19106 (2018).

16. Wang P, *et al.* Atomic insights for optimum and excess doping in photocatalysis: A case study of few-Layer Cu-$ZnIn_2S_4$. *Adv. Funct. Mater.* **29**, 1807013 (2019).

17. Li C, Che H, Huo P, Yan Y, Liu C, Dong H. Confinement of ultrasmall $CoFe_2O_4$ nanoparticles in hierarchical $ZnIn_2S_4$ microspheres with enhanced interfacial charge separation for photocatalytic H_2 evolution. *J. Colloid Interface Sci.* **581**, 764-773 (2021).

18. Zuo G, *et al.* Ultrathin $ZnIn_2S_4$ nanosheets anchored on Ti_3C_2TX MXene for photocatalytic H_2 evolution. *Angew. Chem. Int. Ed.* **59**, 11287-11292 (2020).

19. Zhang Z, Liu K, Feng Z, Bao Y, Dong B. Hierarchical sheet-on-sheet $ZnIn_2S_4$/g-C_3N_4 heterostructure with highly efficient photocatalytic H_2 production based on photoinduced interfacial charge transfer. *Sci. Rep.* **6**, 19221 (2016).

20. Zhuang G, *et al.* Branched In_2O_3 mesocrystal of ordered architecture derived from the oriented alignment of a metal–organic framework for accelerated hydrogen evolution over In_2O_3–$ZnIn_2S_4$. *ACS Appl. Mater. Interfaces* **13**, 9804-9813 (2021).

21. Liu C, Chai B, Wang C, Yan J, Ren Z. Solvothermal fabrication of MoS_2 anchored on $ZnIn_2S_4$ microspheres with boosted photocatalytic hydrogen evolution activity. *Int. J. Hydrogen Energy* **43**, 6977-6986 (2018).

22. Xiong M, Chai B, Yan J, Fan G, Song G. Few-layer WS_2 decorating $ZnIn_2S_4$ with markedly promoted charge separation and photocatalytic H_2 evolution activity. *Appl. Surf. Sci.* **514**, 145965 (2020).

23. Liu H, Zhang J, Ao D. Construction of heterostructured $ZnIn_2S_4$@NH_2-MIL-125(Ti) nanocomposites for visible-light-driven H_2 production. *Appl. Catal., B* **221**, 433-442 (2018).

24. Chao Y, *et al.* $Ni_{1-x}Co_xSe_2$-C/$ZnIn_2S_4$ hybrid nanocages with strong 2D/2D hetero-interface interaction enable efficient H_2-releasing photocatalysis. *Adv. Funct. Mater.* **31**, 2100923 (2021).

25. Ran Q, *et al.* Path of electron transfer created in S-doped NH_2-UiO-66 bridged $ZnIn_2S_4$/MoS_2 nanosheet heterostructure for boosting photocatalytic hydrogen evolution. *Catal. Sci. Technol.* **10**, 2531-2539 (2020).

26. Wang S, Wang Y, Zhang SL, Zang S-Q, Lou XW. Supporting ultrathin $ZnIn_2S_4$ nanosheets on Co/N-doped graphitic carbon nanocages for efficient photocatalytic H_2 generation. *Adv. Mater.* **31**, 1903404 (2019).

27. Li X-l, Wang X-j, Zhu J-y, Li Y-p, Zhao J, Li F-t. Fabrication of two-dimensional Ni_2P/$ZnIn_2S_4$ heterostructures for enhanced photocatalytic hydrogen evolution. *Chem. Eng. J.* **353**, 15-24 (2018).

28. Du C, Yan B, Yang G. Promoting photocatalytic hydrogen evolution by introducing hot islands: SnSe nanoparticles on $ZnIn_2S_4$ monolayer. *Chem. Eng. J.* **404**, 126477 (2021).

29. Chang H, *et al.* Polyoxo-titanium clusters dually functionalized $ZnIn_2S_4$/MIL-101 catalyst for photocatalysis of aquatic hydrogen production. *Int. J. Hydrogen Energy* **45**, 30571-30582 (2020).

30. Jing H, *et al.* Sulfur vacancy-enriched rhombohedral $ZnIn_2S_4$ nanosheets for highly efficient photocatalytic overall water splitting under visible light irradiation. *ACS Applied Energy Materials* **5**, 10187-10195 (2022).

31. Ng B-J, *et al.* Heteroatom P filling activates intrinsic S atomic sites of few-layered $ZnIn_2S_4$ via modulation of H adsorption kinetics for sacrificial agent-free photocatalytic hydrogen evolution from pure water and seawater. *J. Mater. Chem. A*, (2023).

32. Pan R, *et al.* Two-dimensional all-in-one sulfide monolayers driving photocatalytic overall water splitting. *Nano Lett.* **21**, 6228-6236 (2021).

33. Wang Q, *et al.* Oxysulfide photocatalyst for visible-light-driven overall water splitting. *Nature Materials* **18**, 827-832 (2019).

34. Tian B, Gao W, Zhang X, Wu Y, Lu G. Water splitting over core-shell structural nanorod CdS@Cr_2O_3 catalyst by inhibition of H_2-O_2 recombination via removing nascent formed oxygen using perfluorodecalin. *Appl. Catal., B* **221**, 618-625 (2018).
